# CHIR99021, trough GSK-3β Targeting, Reduces Epithelioid Sarcoma Cell Proliferation by Activating Mitotic Catastrophe and Autophagy

**DOI:** 10.3390/ijms222011147

**Published:** 2021-10-15

**Authors:** Sabino Russi, Alessandro Sgambato, Anna Maria Bochicchio, Pietro Zoppoli, Michele Aieta, Alba Maria Lucia Capobianco, Vitalba Ruggieri, Emanuela Zifarone, Geppino Falco, Simona Laurino

**Affiliations:** 1 IRCCS CROB—Referral Cancer Center of Basilicata, 85028 Rionero in Vulture, Italy; sabino.russi@crob.it (S.R.); alessandro.sgambato@crob.it (A.S.); annamaria.bochicchio@crob.it (A.M.B.); pietro.zoppoli@crob.it (P.Z.); michele.aieta@crob.it (M.A.); alba.capobianco@crob.it (A.M.L.C.); vitalba.ruggieri@asl.bari.it (V.R.); emanuela.zifarone@crob.it (E.Z.); simona.laurino@crob.it (S.L.); 2 UOC Clinical Pathology, Altamura Hospital, 70022 Altamura, Italy; 3 Department of Biology, University of Naples Federico II, 80133 Naples, Italy; 4 Biogem—Istituto di Biologia e Genetica Molecolare, 83031 Ariano Irpino, Italy

**Keywords:** autophagy, CHIR99021, epithelioid sarcoma, GSK-3β inhibition, mitotic catastrophe

## Abstract

Epithelioid sarcoma (ES) is a rare disease representing <1% of soft tissue sarcomas. Current therapies are based on anthracycline alone or in combination with ifosfamide or other cytotoxic drugs. ES is still characterized by a poor prognosis with high rates of recurrence. Indeed, for years, ES survival rates have remained stagnant, suggesting that conventional treatments should be revised and improved. New therapeutic approaches are focused to target the key regulators of signaling pathways, the causative markers of tumor pathophysiology. To this end, we selected, among the drugs to which an ES cell line is highly sensitive, those that target signaling pathways known to be dysregulated in ES. In particular, we found a key role for GSK-3β, which results in up-regulation in tumor *versus* normal tissue samples and associated to poor prognosis in sarcoma patients. Following this evidence, we evaluated CHIR99021, a GSK-3 inhibitor, as a potential drug for use in ES therapy. Our data highlight that, in ES cells, CHIR99021 induces cell cycle arrest, mitotic catastrophe (MC) and autophagic response, resulting in reduced cell proliferation. Our results support the potential efficacy of CHIR99021 in ES treatment and encourage further preclinical and clinical studies.

## 1. Introduction

Epithelioid sarcoma (ES) is a rare soft tissue neoplasm described for the first time in the 1970s by Enzinger [1]. ESs represent less than 1% of soft tissue sarcomas (STSs) and are characterized by unclear cellular origin, with both mesenchymal and epithelial features [2,3]. ES mostly affects young adults, mostly males, with a median age of 27 years [4,5,6]. ES can be distinguished by two subtypes that are thought to be a continuum of the same disease: distal- and proximal-type ES [5,7,8]. Prognosis is worse in tumors arising in proximal locations [8] and, as in most cancers, depends on clinical stage, as well as on tumor size, and vascular invasion [6]. Although the majority of patients present with localized disease, recurrence is frequent and, generally, half of patients develop metastasis and have poor outcomes [9]. The cornerstone of localized tumor treatment is complete surgical resection, whereas advanced and metastatic ESs remain usually unresectable and chemoresistant [10]. Radiation therapy combined with surgery is often given to reduce local relapses [11,12]. Although therapeutic regimens based on anthracycline or gemcitabine result in better outcomes compared with all the available chemotherapeutic schedules, the response rates remain poor, being lower than 30% [13,14]. Other administered chemotherapy regimens are single-agents, such as: trabectedin, ifosfamide [15,16], vinorelbine tartrate [17], eribulin [18] or combinations of anthracycline with ifosfamide [15,16,19] or gemcitabine with docetaxel [13,14,19]. Recently, the FDA approved tazemetostat, for the treatment of adults, pediatric and older patients with metastatic or locally advanced epithelioid sarcoma [20]. Notwithstanding advancements in multimodality treatments, the actual therapeutics regimes and, consequently, the ES patient’s survival remains unsatisfactory. A recent study published by Elsamna et al. reported that, at five and ten years from diagnosis, three in five and one in two diagnosed patients will likely survive, respectively [21].

For improving outcomes and overall survival in ES patients, the signaling pathways and mechanisms involved in the progression of ES need to be clarified. From this view, several studies have aimed to better elucidate the molecular deregulations underlying ES pathogenesis. The disruption of pathway regulation by oncogenic mutations or the abnormal expression of key genes promotes tumor onset and progression. As for other kind of tumors, the identification of ES targetable molecular markers and their potential inhibitors will be useful for the development of tailored therapeutic regimens [22]. In this regard, an overexpression of VEGF (vascular endothelial growth factor), an important signaling protein involved in the angiogenesis pathway, has been reported in ES [23]. Moreover, mTOR (mammalian target of rapamycin) signaling hyperactivation has also been observed. Furthermore, mTOR synergic effect with c-MET and EGFR, has been correlated with the development and progression of ES. Several researchers have suggested combinations of c-MET and mTOR or EGFR and mTOR as potential approaches for ES treatment [24,25]. Notably, the complete expression loss of SMARCB1/INI1, one of the best know tumor suppressors, has also been demonstrated in ES. By interacting with several key proteins, it modulates various pivotal pathways [26]. Indeed, its deficiency led to aberrant activation of the WNT signaling pathway, resulting in WNT/β-catenin overexpression [27]. Moreover, SMARCB1/INI1 is known to be a regulator of GLI1 and is reported that its loss causes the Hedgehog–GLI pathway’s aberrant activation [28].

In this study, we combined bioinformatics analyses and in-vitro validation to explore several multitarget chemical inhibitors as potential ES drugs using the cancerRXgene database [29], which contains drug response and genomics data of a large cell lines collection. In particular, we selected the five drugs with the lowest Z-score (representing higher sensitivity) in the VA-ES-BJ epithelioid sarcoma cell line. Among them, we focused on those directed against molecular targets relevant for ES pathogenesis. Following bioinformatics analysis on these drug’s molecular targets, we investigated the effect of GSK-3β inhibition, by CHIR99021, on two different ES cell lines. Interestingly, this drug leads to a strong inhibition of ES cells proliferation through cell cycle arrest, mitotic catastrophe (MC) and autophagy.

## 2. Results

### 2.1. Querying the Genomics of Drug Sensitivity Database for Epithelioid Sarcoma

The Genomics of Drug Sensitivity in Cancer database (GDSC, www.cancerRxgene.org on 30 November 2020) collects datasets that annotate drug sensitivity response in cancer cell lines along with genomic characterization. Using as query VA–ES–BJ, the unique epithelioid sarcoma cell line included in GDSC, we received as output the drugs to which this cell line results sensitive. Among them, we selected five drugs characterized by the highest sensitivity (lowest Z score) and reported to inhibit well-known molecular targets involved in pathogenesis of cancer (Table 1).

While SB505124, palbociclib and parthenolide targets have been suggested as potential actionable biomarkers in ES [30,31], the molecular targets of CHIR99021 and Pevonedistat have not yet been described in epithelioid sarcoma.

### 2.2. GSK-3β Was Associated with Poor Outcome and Was Overexpressed in Tumor versus Normal Sarcoma Samples

**Figure 1:**
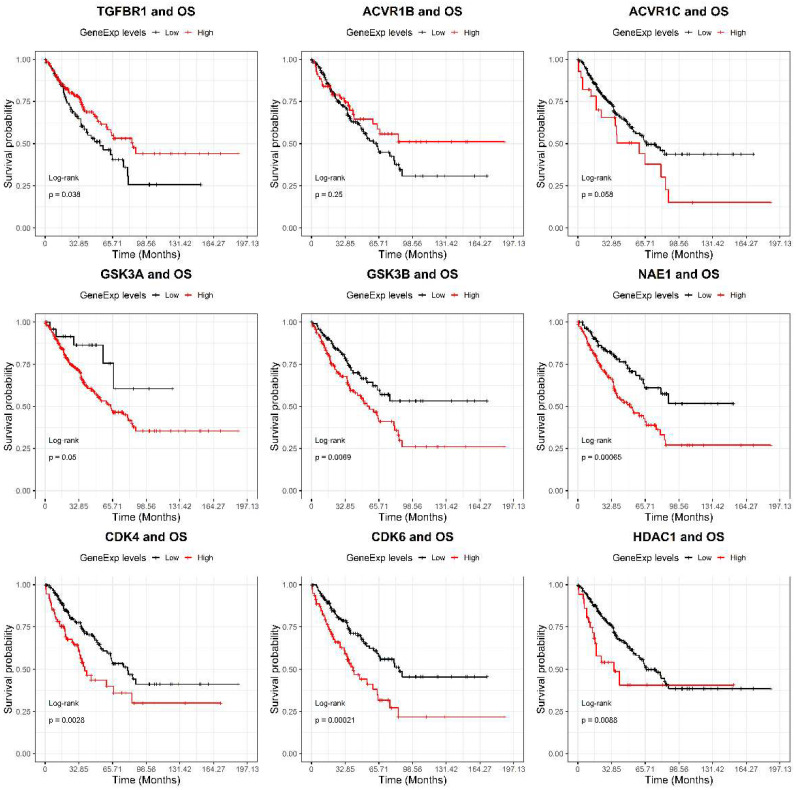
Overall survival (OS) curves, based on mRNA expression levels of the nine drug targets’ genes in the TCGA Sarcoma dataset. High (red) and low (black) expression cohorts were divided through maximally selected rank statistics. The significance of survival differences was estimated by log-rank tests. Number of patients: TGFBR1: low 98, high 161; ACVR1B: low 189, high 70; ACVR1C: low 231, high 28; GSK3A: low 25, high 234; GSK3B: low 117, high 142; NAE1: low 112, high 147; CDK4: low 185, high 74; CDK6: low 179, high 80; HDAC1: low 223, high 36.

In order to select the most suitable chemical compound as potential drug against ES, we evaluated the prognostic value of their target genes. To overcome the rarity of gene expression dataset for epithelioid sarcoma, we performed this analysis using all the soft-tissue sarcoma data included in the TCGA project. Interestingly, we found a strong association of high gene expression levels with poor overall survival (OS) for GSK3B (HR: 1.7 *p* = 0.008), NAE1 (HR: 2.1 *p* = 0.0008), CDK4 (HR: 1.8 *p* = 0.003), CDK6 (HR: 2.1 *p* = 0.0003) and HDAC1 (HR: 2.0 *p* = 0.01) genes (Figure 1).

**Figure 2:**
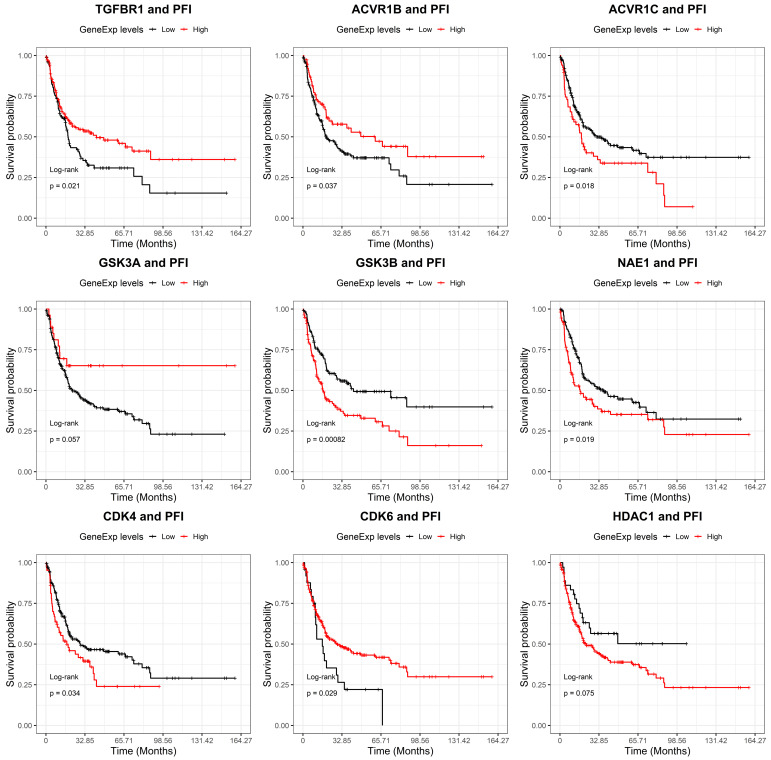
Progression-free interval (PFI) curves, based on mRNA expression levels of the nine drugs’ target genes in the TCGA Sarcoma dataset. High (red) and low (black) expression cohorts were divided through maximally selected rank statistics. The significance of survival differences was estimated by log-rank tests. Number of patients: TGFBR1: low 104, high 155; ACVR1B: low 182, high 77; ACVR1C: low 191, high 68; GSK3A: low 232, high 27; GSK3B: low 121, high 138; NAE1: low 149, high 110; CDK4: low 195, high 64; CDK6: low 25, high 234; HDAC1: low 36, high 223.

Moreover, we investigated the associations of the drugs’ target-genes expression with the progression-free interval (PFI). A significant relation arisen for ACVR1C (HR: 1.5 *p* = 0.02), GSK3B (HR: 1.8 *p* = 0.001) and NAE1 (HR: 1.5 *p* = 0.02) highlighting a negative impact of high gene expression levels on PFI (Figure 2).

Notably, only two genes, GSK3B and NAE1, showed a concordant behavior between OS and PFI.

Moreover, we evaluated, by log rank test and multivariate cox analysis, the overall and the progression free survival associated with GSK3B and with the following clinical features: age, gender, histology, and radiotherapy. Interestingly, both full models (OS and PFI) were significant (*p* < 0.05) due to the contribution of higher GSK3B gene-expression levels. In addition to GSK3B, only age resulted in significant (*p* < 0.05) association with OS in both uni- and multivariate analyses. According to an Akaike information criterion (AIC)-based stepwise procedure, GSK3B expression alone (with age only with the OS) better models overall and progression-free survival. In particular, for both OS and PFI, the global *p*-value (log-rank) of the model was <0.001 with hazard ratios (HRs) 1.93-fold and 1.81-fold greater for GSK3B, in the OS and PFI models, respectively. Although significant, age HR was low (1.02-fold) in the OS model. Finally, GSK3B turned out to be an independent prognostic factor for both PFI and (practically) OS.

To verify whether the nine target genes were up-regulated in sarcoma samples versus normal tissue specimens, we compared their expression levels using GEPIA2 on-line tool (gepia2.cancer-pku.cn, accessed on 1 September 2021) [32].

**Figure 3:**
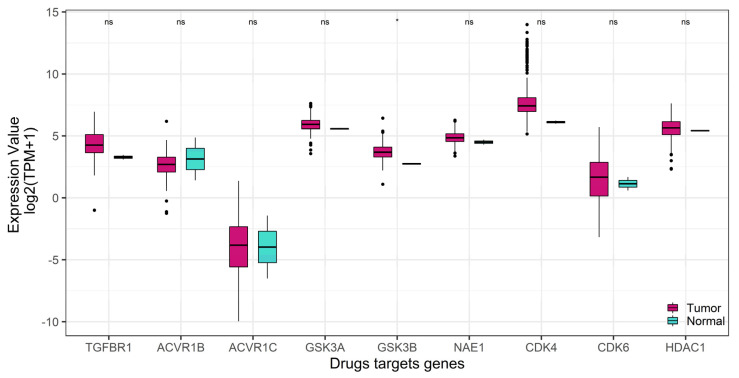
Expression of the nine target genes in tumor and normal samples (TCGA Sarcoma dataset). Expression values of genes are log_2_–transformed. Statistical significance was assessed by ANOVA. Number of patients: tumor = 262, normal = 2.

Remarkably, as shown in Figure 3, only GSK3B resulted in significant overexpression in tumor samples. This finding is in accordance with the poor OS (Figure 1) and PFI (Figure 2) associated with high GSK3B expression.

Overall, based on prognostic values in sarcoma patients, overexpression in soft tissue sarcomas as compared with normal tissues and the sensitivity of the epithelioid sarcoma cell line to CHIR99021, we considered GSK-3β a potential drug target worthy of further investigation.

### 2.3. Inhibition of GSK-3β Induces an Anti-Proliferative Effect on Epithelioid Sarcoma Cells

In order to validate GSK-3β as a cancer target, we characterized the effects of its inhibitor, CHIR99021 (Figure S1A), on VA-ES-BJ cell line. CHIR99021 is a potent (Ki values of 9.8 nM) and selective inhibitor of GSK-3α/β [33], resulting in the alteration of its downstream targets. However, to date, there are no reports on the effects of CHIR99021 on epithelioid sarcoma cells.

**Figure 4:**
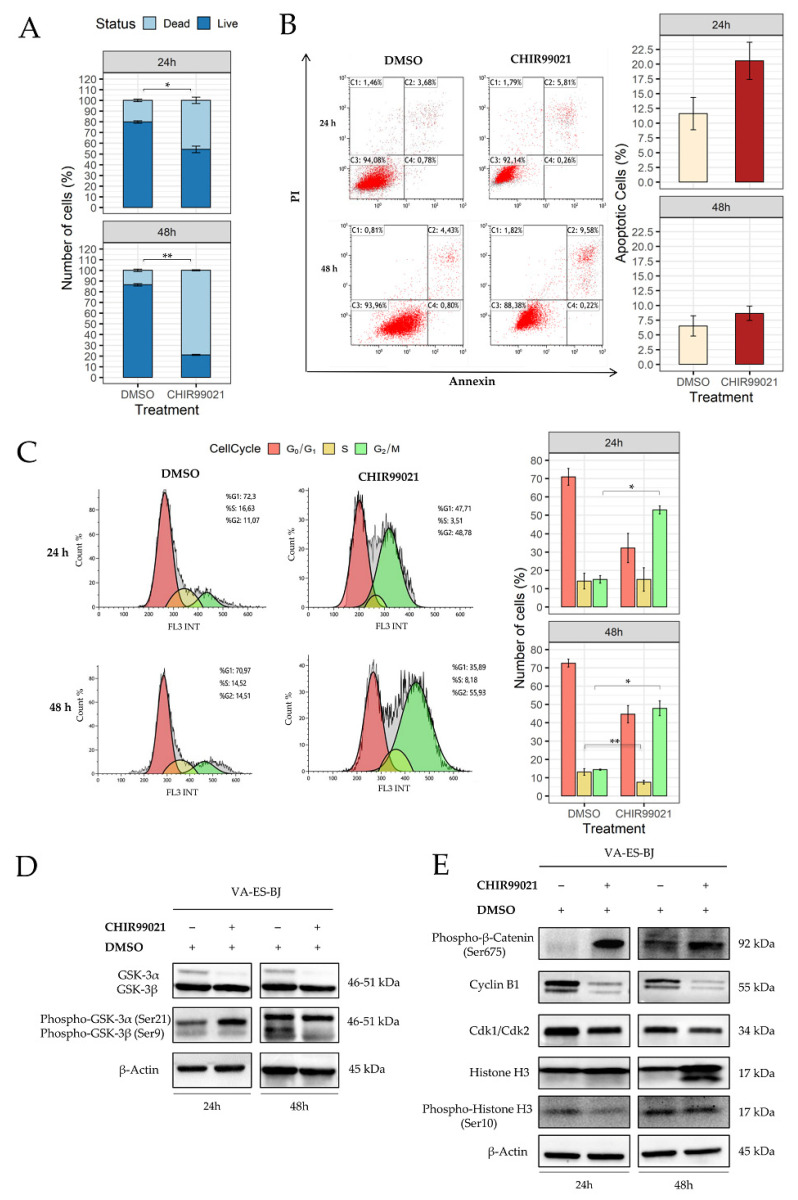
CHIR99021 affects the proliferation of as ES cell line. (**A**) CHIR99021’s effect on the viability of the VA-ES-BJ cell line evaluated using a trypan blue exclusion assay. Cell line was exposed to 100 µM for 24 and 48 h. Cell viability is presented as percentages distinguishing dead cells (light blue) from live cells (blue). The graph represents means ± SE of three independent experiments; differences between treatments groups were estimated by paired *t* tests. (**B**) Dot plot diagrams following 24 and 48 h treatments of VA-ES-BJ cells with CHIR99021 analysed by FACS, after Annexin V-FITC and PI labelling. Representative dot plots present intact cells in the lower-left quadrant, early apoptotic cells in the lower-right quadrant, late apoptotic or necrotic cells in the upper-right quadrant and necrotic cells in the upper-left quadrant. Histogram reported the mean ± SE of apoptotic cell percentage from three separate experiments. (**C**) Representative plots of DNA content distribution of cell cycle phases of VA-ES-BJ cell treated with CHIR99021 100 µM for 24 and 48 h. Histogram represents the percentage of cell number in the G_0_/G_1_, S, and G_2_/M phases after treatment. The values are representative of means ± SE of three separate experiments using paired *t* tests (*: *p* < 0.05; **: *p* < 0.001). (**D**) Western blot analysis showing the expression of GSK-3α/β and *p*-GSK-3α/β proteins in VA-ES-BJ cell after treatment with CHIR99021 or vehicle for 24 and 48 h. The same blot was reprobed with anti-β-Actin to confirm the equal loading of each lane (40 μg of whole lysate). (**E**) Western blot analysis reported the expression levels of protein related to cell cycle signals at 24 and 48 h after CHIR99021 100 µM treatment. The blot was first incubated with anti-Cdk1/Cdk2, anti-CyclinB1, anti-phospho-HistoneH3, anti-HistoneH3, anti-phospho-β-Catenin and then reprobed with anti-β-Actin to confirm the equal loading of each lane (40 μg of whole lysate).

To elucidate the consequences of CHIR99021 exposure, we evaluated anti-proliferative effect performing a MTS assay at different concentrations and time points. We determined the IC50 value by treating cells with seven different concentrations, ranging from 2.5 µM to 150 µM for 24, 48 and 72 h. CHIR99021 demonstrated a dose-dependent effect on viability of VA-ES-BJ cell line, being significant at 100 µM. In contrast, no time effect was observed (Figure S1B). Therefore, we used this IC50 value to perform subsequent experiments. Subsequently, to confirm CHIR99021 effects on VA-ES-BJ cells’ viability, we carried out a trypan blue exclusion assay. As shown in Figure 4A, we found a significant decline of live cells number after CHIR99021 treatment compared with vehicle (DMSO) values. In particular, after 24 h of treatment, live cells significantly decreased from 79.9% ± 1.2 to 54.2% ± 3.0 (DMSO vs. CHIR99021, *p* = 0.04).

A similar trend was confirmed, also; after 48 h of treatment, a more significant decrease of live cells (86.5% ± 1.2 vs. 20.9% ± 0.5; DMSO vs. CHIR99021 *p* = 0.0008) was observed.

To investigate whether the anti-proliferative effect of CHIR99021 was due to apoptosis or cell cycle arrest, we performed flow cytometry evaluations.

As shown in Figure 4B, no significant differences in apoptosis were observed both after 24 h and 48 h of treatment with CHIR99021 100 µM on VA-ES-BJ cell line. In particular, after 24 h of treatment we measured the following apoptosis rates: CHIR99021 20.5% ± 3.2; DMSO 11.6% ± 2.7. Similarly, after 48 h of treatment we observed 8.6% ± 1.2, and 6.5% ± 1.7 of apoptotic cells for CHIR99021 and DMSO, respectively. These data highlighted that the inhibition of cell proliferation by CHIR99021 did not correlate with apoptosis.

Conversely, cell cycle analysis emphasized a strong accumulation of treated cells in the G_2_/M phase increasing significantly from 15.0% ± 2.0 (DMSO) to 52.8% ± 2.2 (CHIR99021) (*p* = 0.02). No differences were recorded for the S and G_0_/G_1_ phases at 24 h. Cell cycle analysis at 48 h revealed an increased percentage of G_2_/M cells varying from 14.4% ± 0.3 (DMSO) to 47.9 ± 4.1 (CHIR99021) (*p* = 0.01). Moreover, a concomitant decrease of cells at S phase was detected (13.1% ± 1.8, DMSO vs. 7.5% ± 1.0 CHIR99021; *p* = 0.0005) (Figure 4C).

To examine whether this cell-cycle-perturbing effect of CHIR99021 could also occur in non-cancer cells we used HDFa cells. Indeed, by sharing the mesenchymal origin with epithelioid sarcoma [4], they can represent a suitable non-cancer counterpart.

The results suggested that CHIR99021 impairs the cell cycle progression of ES cancer cells, resulting in it’s being well-tolerated by normal cells (Figure S2).

Finally, we evaluated, by Western-blotting analysis, the expression levels of CHIR99021’s own targets, GSK-3α/β, as well as β-catenin, one of the main downstream signaling molecules.

As reported in Figure 4D, Western-blotting analyses revealed a decrease of both GSK-3α/β isoforms, more evident for GSK-3α, and an increase of phospho-GSK-3α.

This was accompanied by a simultaneous increase of β-Catenin phosphorylated at Ser675, the transcriptionally active form [34]. Interestingly, accumulation of endogenous β-Catenin is distinctive of G_2_/M cell cycle phase [35] (Figure 4E). Hence, we confirmed cell cycle arrest by measuring the key driver of cell cycle progression: CyclinB1 and its partner, Cyclin-dependent kinase 1 (Cdk1), the activation of which has an important role in mitosis entry. We found reduced levels of both Cyclin B1 and Cdk1 that clearly indicate an inhibition of complex formations and a cell cycle arrest in the G_2_/M phase [36].

To better clarify whether CHIR99021 induces G_2_ or M arrest, we measured the expression of Histone H3 and its phosphorylated form, both hallmarks of mitosis. Our data showed increased levels of these specific mitotic markers (Figure 4E).

### 2.4. GSK-3β Inhibition Induces Cell Morphology Aberration Resembling Mitotic Catastrophe

Under light filed microscopy evaluation, we observed a heavy alteration of cell morphology after CHIR99021 treatment. Since literature data reported that prolonged cell cycle arrest in G_2_/M phase could evolve in mitotic catastrophe (MC), we evaluated the presence of giant micronucleate cells, a main feature of MC, by fluorescent staining of nuclei in VA-ES-BJ cell line exposed to CHIR99021.

**Figure 5:**
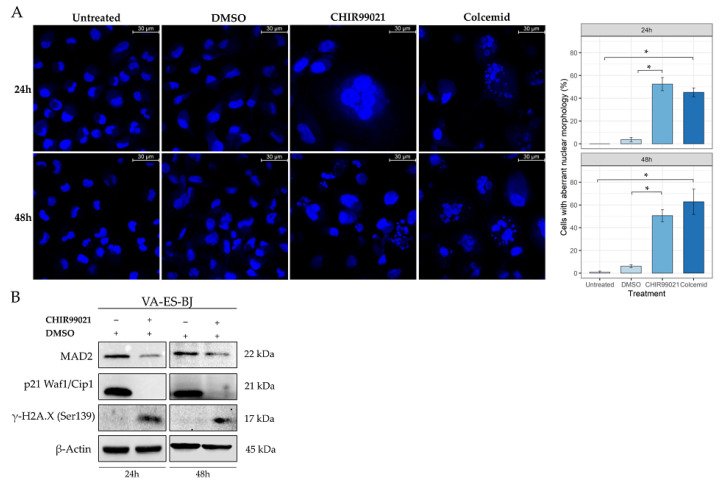
CHIR99021 induces mitotic catastrophe in the ES cell line. (**A**) Cells were treated with CHIR99021 100 µM for 24 or 48 h, stained with Hoechst-33342, and examined under fluorescence microscopy at 63X magnification. As shown, many cells have characteristics peculiar of mitotic catastrophe. The aberrant morphology of cells was based on the evaluation of at least 30 nuclei in each sample. Each bar is the mean ± S.E. of three separate experiments; * *p* < 0.05, versus the relative control. (**B**) Western blot analysis reported the expression levels of protein related to mitotic or spindle checkpoint arrest at 24 and 48 h after CHIR99021 100 µM treatment on VA–ES–BJ. The blot was first incubated with anti-MAD2, anti-p21 Waf1/Cip1 and anti-γ-H2A.X and then reprobed with anti-β-Actin to confirm the equal loading of each lane (40 μg of whole lysate).

We treated VA-ES-BJ cells with CHIR99021 100 µM or Colcemid 1 µg/mL, the latter by interfering with correct microtubule polymerization and mitosis completion represented the positive control. As shown in Figure 5A, after 24 h of treatment, we observed numerous cells with specific MC-related morphology both for CHIR99021 (52.4% ± 5.8; *p* = 0.008) and Colcemid (45.2% ± 3.9; *p* = 0.007) as compared with vehicle (3.7% ± 1.9) or untreated controls (0.0% ± 0.0), respectively.

The same scenario, large cells with multiple micronuclei, resulted also after 48 h of treatment: CHIR99021 (50.6% ± 5.3; *p* = 0.01) vs. DMSO (6.2% ± 1.3) and Colcemid (62.8% ± 11.1; *p* = 0.003) vs. untreated (0.9% ± 0.9). No significant difference was observed between CHIR99021 and Colcemid demonstrating a comparable effectiveness of these two compounds.

In addition, we detected a strong reduction of the MAD2 protein’s expression levels, a key factor involved in mitotic spindle checkpoint.

Moreover, we revealed the reduction of p21 protein levels, another key factor required for the correct regulation of centrosome amplification, and, concomitantly, increased levels of γ-H2A.X, which were reported to occur in mitotic cells (Figure 5B). This evidence supports the idea of a mitotic machinery impairment that led to an alteration of cell cycle progression.

### 2.5. Autophagy Response as the Cell Death Mechanism Mediated by GSK3-β Inhibition

**Figure 6:**
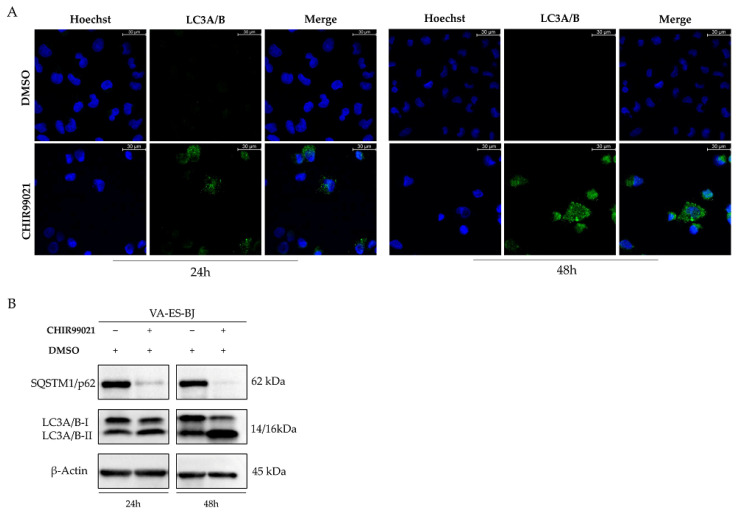
CHIR99021 induces autophagy in the VA-ES-BJ cell line. Cells were treated with CHIR99021 100 µM for 24 or 48 h and detecting LC3A/B levels by immunofluorescences. (**A**) Increase of LC3-positive puncta formation supported the involvement of autophagy in VA-ES-BJ cells. (**B**) Western blot analysis reporting the expression levels of protein related to autophagic process at 24 and 48 h after CHIR99021 100 µM treatment. The blot was first incubated with anti-LC3A/B and then reprobed with anti-β-Actin to confirm the equal loading of each lane (60 μg of whole lysate).

Since we excluded an apoptotic cell death after CHIR99021 treatment, we hypothesized an autophagic response as a possible mechanism of cell death consequent to MC [37]. Therefore, we evaluated the expression levels of autophagy regulators and marker proteins. Interestingly, we revealed a strong autophagy response, as evidenced by an increased conversion of LC3A/B-I to LC3A/B-II in CHIR99021-treated cells (Figure 6A,B). These findings were also supported by a strong decrease of p62 after treatment (Figure 6B).

**Figure 7:**
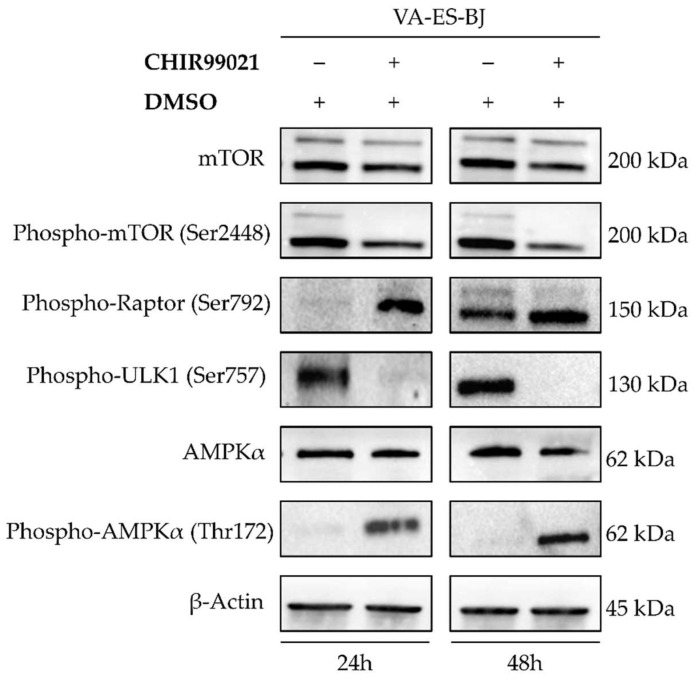
CHIR99021 promotes autophagy through AMPK/mTORC1/ULK1 axis. Western blots showing levels of specific autophagy markers in VA-ES-BJ cells measured at 24 and 48 h after exposure to CHIR99021 100 µM. The blot was first incubated with anti-mTOR, anti-phospho-mTOR, anti-phospho-RAPTOR, anti-AMPK, anti-phospho-AMPK, anti-phopho-ULK1 and then reprobed with anti-β-Actin to confirm the equal loading of each lane (60 μg of whole lysate).

Furthermore, to clarify the molecular mechanisms underlying the autophagic response consequent to GSK-3β inhibition, we investigated whether CHIR99021 treatment led to AMPK activation, one of the main upstream regulators of autophagy pathway and at the same time a direct target of GSK3-β. We observed a marked increase of AMPK phosphorylation at residue Thr172. Consequently, 24 h after 100 µM CHIR99021 treatment, we detected phosphorylation of mTORC1 component Raptor at Ser792 that lead to mTORC1 inhibition, confirmed, also, by a reduction of phospho-mTOR levels. To settle this mechanism of action, we evaluated the phosphorylation of the mTOR downstream target ULK1 at Ser757, finding a strong reduction in its levels (Figure 7).

### 2.6. Evaluation of CHIR99021 Effects on the NEPS Cell Line

To strengthen our results, thanks to the collaboration with Prof. Hiroyuki Kawashima, we replicated the key experiments on NEPS cells, a cell line derived from distal-type ES.

**Figure 8:**
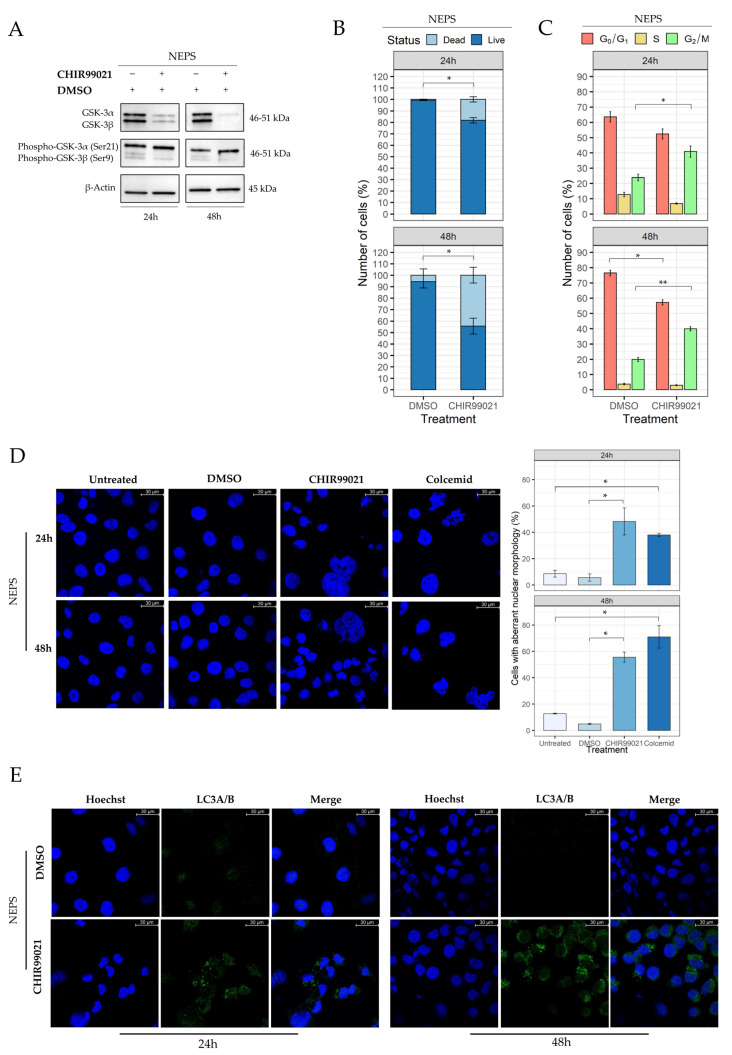
CHIR99021 affects the proliferation of the NEPS cell line. (**A**) Western blot analysis showing the expression of GSK-3α/β and phospho-GSK-3α/β proteins in NEPS cells after treatment with CHIR99021 or vehicle for 24 and 48 h. The same blot was reprobed with anti-β-Actin to confirm the equal loading of each lane (40 μg of whole lysate). (**B**) CHIR99021 effect on the viability of the NEPS cell line, evaluated using a trypan blue exclusion assay. Cells were exposed to 50 µM for 24 and 48 h. Cell viability is presented as percentages distinguishing dead cells (light blue) from live cells (blue). The graph represents means ± SE of three independent experiments; differences between treatments groups were estimated by paired *t* tests. (**C**) Distribution of cell cycle phases of NEPS cell treated with CHIR99021 50 µM for 24 and 48 h. Histogram represents the percentage of cell number in the G_0_/G_1_, S, and G_2_/M phases after treatment. The values are representative of means ± SE of three separate experiments using paired t test (*: *p* < 0.05; **: *p* < 0.001). (**D**) CHIR99021 induces mitotic catastrophe in the NEPS cell line. Cells were treated with CHIR99021 50 µM for 24 or 48 h, stained with Hoechst-33342, and examined with fluorescence microscopy at 63X magnification. The aberrant morphology of cells was based on the evaluation of at least 30 nuclei in each sample. Each bar is the mean ± S.E. value from three separate experiments; (* *p* < 0.05, ** *p* < 0.001 versus the relative controls. (**E**) Cells were treated with CHIR99021 50µM for 24 or 48 h and LC3A/B-fluorescent puncta were detected.

Through MTS assay, we determined the IC50 value as carried out for the VA-ES-BJ cell line. Interestingly, the NEPS cell line resulted in a sensitivity to a lower dose of CHIR99021 (50 µM), also confirming the absence of time-dependent effects (Figure S1C). Western blotting analysis, in Figure 8A, revealed a decrease of both GSK-3α and β isoforms, more evident in the NEPS cells relative to VA-ES-BJ cells. An increase of phospho-GSK-3α, confirming the specificity of CHIR99021, was also observed. Notably, since the basal level of GSK-3α/β appears different between the two cell lines, we can speculate that the higher GSK-3α/β level in VA-ES-BJ cell line justifies the higher IC50 dose needed to inhibit cell proliferation.

As reported in Figure 8B, the trypan blue exclusion assay showed a reduction of live cells which were 81.7% ± 2.3 after 24 h of CHIR99021 treatment as compared with vehicle control 99.17% ± 0.24 (*p* = 0.01). After 48 h, live cells were 55.6% ± 6.9 vs. 94.4% ± 5.6 (treated vs. vehicle, *p* = 0.01).

Cell cycle alteration was also confirmed in the NEPS cell line, in which we found a higher number of cells in the G_2_/M phase after CHIR99021 at 50 µM compared with control (DMSO) (40.8% ± 3.6 vs. 23.8% ± 2.2 *p* = 0.02). No differences were recorded for S and G_0_/G_1_ phase at 24 h. The same scenario was observed at 48 h, being cells in G_2_/M phase 39.8% ± 1.4 and 19.8% ± 1.4 for CHIR99021 and vehicle, respectively (*p* = 0.0005). Significant difference was observed in G_0_/G_1_ phase at 48 h (CHIR99021 57.2% ± 1.7 vs. DMSO 76.5% ± 1.9; *p* = 0.002) (Figure 8C).

As previously observed for VA-ES-BJ cell line, MC involvement was highlighted under fluorescence microscopy also in NEPS cell line using its CHIR99021 IC50 value (50 µM). In particular, after 24 h of treatment, cells with MC-related features accounts for: 48.2% ± 10.2 (CHIR99021; *p* = 0.03) vs. 5.6% ± 2.8 (DMSO) and 37.9% ± 1.1 (Colcemid; *p* = 0.004) vs. 8.5% ± 2.6 (untreated). Similarly, after 48 h we found: 55.6% ± 3.8 (CHIR99021; *p* = 0.006) vs. 4.9% ± 0.45 (DMSO) and 71.0% ± 8.5 (Colcemid; *p* = 0.02) vs. 12.8% ± 0.3 (untreated) (Figure 8D).

The setting of an autophagy response was confirmed in NEPS cells by the evidence of LC3A/B-fluorescent puncta (Figure 8E). These data support our hypothesis that GSK-3β inhibition by CHIR99021 has a key role in inducing MC- and autophagy-mediated cell death.

## 3. Discussion

To date, clinically helpful molecular targets for soft tissue sarcomas are still lacking, and even more so for epithelioid sarcoma. Moreover, due to the rarity of this tumor and poor long-term outcomes, little is known about the response to systemic treatments in ES patients and this knowledge scarcity highlights that current chemotherapy regimens are inadequate. Indeed, literature on ES treatment outcomes is limited to case reports or studies with a poor number of enrolled patients.

Novel systemic treatments for ES remain a crucial objective to be pursued with high priority. In this regard, a major limit for research focused on this rare disease is the lack of patients’ samples, in-vitro and in-vivo models, high-throughput data, and large clinical trials. Given that, current ES treatments seems to be only an extended application of drugs effective in other kinds of tumors but not tailored for epithelioid sarcoma.

In this scenario, we aimed to identify new possible molecular targets and their respective pharmacological inhibitors through an innovative approach considering the molecular features of ES.

We first queried the GDSC database, which offers a large drug sensitivity dataset that contributes to discovery of new therapeutic biomarkers for cancer treatments. Among the outputted drugs, we selected those to which the VA-ES-BJ cell line resulted sensitive and those that target signaling pathways dysregulated in this type of cancer. Then, through bioinformatics analyses performed on data from TCGA Sarcoma project, we restricted the in-vitro evaluation to the drugs whose molecular targets’ gene expressions were associated with poor prognosis and were overexpressed in cancer versus normal tissues.

GSK-3β resulted the only target gene satisfying the above-mentioned selection criteria, so we used CHIR99021, a selective inhibitor of GSK-3α/β, to investigate whether it could represent a suitable drug for the treatment of epithelioid sarcoma. GSK-3β is a fascinating enzyme, on which the literature is immense and quite often provides conflicting statements and observations. GSK-3β plays pivotal roles in the pathogenesis of several diseases, including cancer [38,39]. It is a serine/threonine kinase able to phosphorylate a large number of substrates, assuming different roles in either promoting or repressing cancer-cell survival, proliferation and invasion in several type of cancers [40,41,42], including sarcomas [41,43,44]. We evaluated and demonstrated, for the first time, that the inhibition of GSK-3β has an effect on epithelioid sarcoma cells’ proliferation. We strengthened our findings by using two cancer cell lines derived from the two different epithelioid sarcoma subtypes: distal and proximal. In particular, we found different IC50 values for the two cell lines, which reflect basal levels of GSK-3α/β. On such basis, we can speculate that an association between GSK-3α/β levels and aggressiveness could exist. Furthermore, our results highlighted the involvement of mitotic catastrophe-linked cell death evolving in autophagy.

Indeed, we confirmed a reduction of cell proliferation by MTS assay and an accumulation of cells in G_2_/M phase after CHIR99021 treatment by flow cytometry. It is well known that G_2_/M transition is mediated by the activation of the CyclinB1/Cdk1 complex, which activity favors the entry in mitosis [45,46]. Accordingly, our results showed a down-regulation of CyclinB1 and Cdk1 protein levels following treatment with CHIR99021. G_2_/M arrest was also supported by the increase of β-Catenin and phospho-Histone H3 (Ser10) protein levels, the latter playing a pivotal role in the regulation of mitotic catastrophe [47]. Indeed, microscopy observation revealed the typical pattern of MC, a process resulting from aberrant mitosis, characterized by the formation of multinucleated cells and leading to cell death [48]. Treatment of ES cells with CHIR99021 resembled the MC unique features obtained following Colcemid treatment, an anticancer agent known to interfere with microtubule polymerization that consequently determines a mitotic arrest [49,50].

Notably, we also found a strong reduction of p21 protein level supporting the idea that cells normally progress from G_2_ to M phase but did not complete mitotic division due to delaying the association between Cdk1 and CyclinB1 [51,52].

Moreover, we observed a concomitant reduction of MAD2, as well as an increase of γ-H2A.X levels. These findings strongly reinforce, in line with a previous report [53], the hypothesis that ES cells arrest in the mitotic phase following CHIR99021 treatment. Analyzing the MC consequences, we discovered that CHIR99021 treatment caused the activation of autophagy pathways, finding a link between MC and autophagic cell death. This link was confirmed evaluating, by Western-blotting and immunofluorescence experiments, LC3A/B expression, a specific marker of autophagy. LC3A/B distribution pattern is typically diffuse in normal conditions (LC3-I) becoming punctate when cleaved in the LC3-II form during an autophagic process [54], as we clearly observed after CHIR99021 treatment. This evidence was reinforced by a strong reduction of p62 [55].

In order to define the signaling pathway responsible for triggering autophagy subsequent to GSK-3β inhibition, we firstly studied the possible cross-talk between GSK-3β and an autophagy upstream pathway regulator. Previous reports showed that GSK-3β negative regulation of AMPK activity has a fundamental role in autophagy control [56], so we determined the levels of AMPK phosphorylation at Threonine 172; an essential prerequisite for AMPK activity. The high levels of AMPK in the phosphorylated form allowed us to speculate a negative feedback between GSK-3β and AMPK in the VA-ES-BJ cell line, in which phospho-AMPK directly phosphorylates Raptor, a component of mTORC1 complex [57]. According with this molecular mechanism, our results showed an increase of phospho-Raptor, the phosphorylation of which determines mTORC1’s inactivation [58,59]. Further, we found, after both 24 h and 48 h of CHIR99021 treatment, a concomitant decrease of mTOR and even more a marked decrease of its active form, phospho-mTOR. Finally, we evaluated the phosphorylation of ULK1, a key effector of the mTORC1-dependent regulation of autophagy [60,61,62]. Indeed, previous studies reported that inhibition of mTORC1 leads to a low phosphorylation of ULK1 at Ser757 with consequent activation of autophagy cascade. Our results are in line with all this evidence and support the key role of CHIR99021 in mediating autophagy activation via AMPK/mTORC1/ULK1 in epithelioid sarcoma cells.

In this study, we took a step back, starting from an in-vitro model to find new drugs selective for ES treatment. We also aimed to encourage the scientific community to move in this direction, in particular for those rare diseases that have not yet found optimal treatments capable of improving patients’ outcome.

## 4. Materials and Methods

### 4.1. Cell Culture and Maintenance

The human cell lines used for this study were: VA-ES-BJ (ATCC^®^ CRL2138™), NEPS, and Primary Dermal Fibroblast; Normal, Human, Adult (HDFa). The VA-ES-BJ and HDFa cell lines were purchased from the American Type Culture Collection (ATCC), the NEPS cell line was provided by NU’s researcher; Hiroyuki Kawashima MD, PhD/Professor/Division of Orthopedic Surgery, Niigata University Graduate School of Medical and Dental Sciences [63]. VA-ES-BJ was grown in Dulbecco’s modified Eagle’s medium (DMEM, GIBCO, Grand Island, NY, USA) with 4.5 g/L glucose supplemented with 10% heat-inactivated Fetal Bovine Serum (FBS; GIBCO, Grand Island, NY, USA), and 0.1 mM non-essential amino acids (MEM, GIBCO, Grand Island, NY, USA). HDFa (ATCC^®^ PCS201012™) was cultured in Fibroblast Basal Media (ATCC PCS-201-030) supplemented with Fibroblast Growth Kit components (ATCC PCS-201-040). Cell lines were maintained in a humidified incubator at 37 °C and 5% CO_2_; however, they were passed no more than 4–10 times, after which, we again utilized cells from original stocks. NEPS cells were cultured in Roswell Park Memorial Institute 1640 medium (RPMI 1640, GIBCO, Grand Island, NY, USA) supplemented with 10% FBS and 1% penicillin–streptomycin and 1% l-glutamine. All cell lines were tested for mycoplasma infection upon receipt using LookOut^®^ Mycoplasma PCR Detection Kit (Sigma-Aldrich, Saint Louis, MO, USA).

### 4.2. Drugs Data Analysis: Inhibitors Selection

Starting from the cancerRXgene database data, which contains drug response data for different cell lines, we selected the five drugs with the lowest Z scores, which represents the number of standard deviations from the mean of IC50 values (standardization) [29].

### 4.3. Cell Viability Assays

We performed a sensitivity assay using CHIR99021 (GSK-3α and GSK-3β inhibitor), which was purchased from Cayman Chemical (Ann Arbor, MI, USA). The stock solution was obtained resuspending the lyophilized form in dimethyl sulfoxide (DMSO, Sigma-Aldrich, Saint Louis, MO, USA) and then diluted in fresh medium to the desired concentrations. The maximum DMSO volumes used were always <2%.

Cells were plated at a density of 3 × 10^3^ cells/well into 96-well plates and grown overnight before treatments with the inhibitor, or vehicle for 24, 48 and 72 h. Cells’ viabilities were assessed using MTS assay. A solution of CellTiter 96^®^ Aqueous MTS Reagent Powder (Promega, Madison, WI, USA) was added to each well. After incubation of 1.5 h at 37 °C the absorbance at 490 nm was measured and the percentage of viability in each well was calculated using the untreated cells as 100%. Three independent measurements were performed in triplicate for each assay. The CHIR99021 concentration effective to reduce cell proliferation by 50% (IC50) was estimated following the dose-response curves carried out using seven points, ranging from 2.5 to 150 μM.

The trypan blue dye exclusion assay was also performed to determine the number of viable cells. Briefly, 10 μL of control and treated cell suspensions were added to 10 μL of trypan blue dye (Sigma-Aldrich, Saint Louis, MO, USA) and 10 μL of this solution was loaded in a Burker chamber and counted under 10× magnification using an inverted microscope. The results were expressed as the percentage of live and dead cells for each condition.

### 4.4. Cell Apoptosis Assay

VA-ES-BJ cells were seeded in 25 cm^2^ flask and cultured to 70% confluence prior to be exposed with CHIR99021 100 μM or control media, containing an equivalent amount of DMSO, for 24 and 48 h. Briefly, cells were harvested and after washing twice in cold PBS were centrifuged at 1500 rpm at 4 °C and resuspended in 100 μL of binding buffer. Cells were double-stained with 5 μL FITC-Annexin V and 5 μL propidium iodide (BD Biosciences, Franklin Lakes, NJ, USA) and then incubated for 15 min at room temperature in the dark. Number of apoptotic cells was detected using a Navios flow cytometer (Beckman Coulter, Miami, FL, USA). Data were analyzed by Kaluza analysis software 2.1.

### 4.5. Cell Cycle Assay

VA–ES–BJ, NEPS and HDFa cells were seeded at 2.5 × 10^5^ cells in 25 cm^2^ culture flask. At 24 h after seeding, the cells were washed with PBS, and then CHIR99021 at 100 μM or 50 μM or DMSO was added. After 24 and 48 h, cells were harvested, washed and fixed in ice-cold 70% ethanol for 1 h. Fixed cells were then incubated with PI/RNaseA staining solution for 30 min at room temperature and dark. A total of 1 × 10^4^ events were acquired by Navios flow cytometer performing the analyses using the Kaluza 2.1 software.

### 4.6. Western Blot Analysis

Cells were harvested and the pellets were incubated in RIPA lysis buffer (Thermo Scientific, Waltham, MA, USA) containing protease and phosphatase inhibitors for 30 min on ice. Subsequently, at least 40 μg of extracted proteins were electrophoresed through 4–20% poly-acrylamide gels. After transferring the proteins to PVDF membranes using a TransBlot Turbo system (Bio-Rad, Hercules, CA, USA), the membranes were blocked with 5% non-fat milk in PBS with 0.1% TWEEN^®^ 20 for 1 h and then incubated separately with primary antibodies at 4 °C overnight. Membranes were then incubated with HRP-conjugated secondary antibody and stained with an enhanced chemiluminescence kit (Clarity Western ECL Substrate, Bio-Rad). The signals were acquired by ChemiDoc Imaging System XRS + (Bio-Rad). In order to detect the expression levels of GSK-3α/β, its signaling-related proteins were used: GSK-3α/β (CST, Cat#5676), phospho-GSK-3α/β (CST, Cat#5558) and rabbit monoclonal anti-phospho-β-Catenin (Ser675) (CST, Cat#8814). To detect the expression levels of cell cycle-related proteins, the following antibodies from Cell Signaling Technology (Boston, MA, USA), Santa Cruz Biotechnology (Dallas, TX, USA) and Abcam (Cambridge, UK) were used: Cdk1/Cdk2 (sc-53219), Cyclin B1 (CST, Cat#4138) HistoneH3 (CST, Cat#4499) and phospho-HistoneH3 (Ser10) (CST, Cat#9701). Furthermore, to evaluate the expression levels of the mitotic spindle check-point proteins MAD2 (sc-374131), the anti-gamma-H2A.X (ab-11174) and p21 Waf1/Cip1 (12D1) (CST, Cat#2947) antibodies were used. Finally, to detect autophagy marker expression and autophagy signaling related proteins the following antibodies were employed: LC3A/B (CST, Cat#4108), SQSTM1/p62 (CST, Cat#5114), AMPK (CST, Cat#2532), phospho-AMPK (Thr172) (CST, Cat#2535), mTOR (sc-517464), phospho-mTOR (ser2448) (ab-1093), phospho-Raptor (Ser792) (CST, Cat#2083) and phospho-ULK (ser757) (CST, Cat#14202). Mouse monoclonal anti-β-Actin (CST, Cat #4970) antibody was used as a loading control.

### 4.7. Immunofluorescence and Confocal Microscopy Analysis

About eighty-thousand cells were seeded on chamber slides and cultured for 24 and 48 h after CHIR99021 or DMSO treatment. A solution of Colcemid at 1 µg/mL was used as positive control to detected mitotic arrest. Cells were fixed with 4% (*w/v*) formaldehyde in PBS at room temperature for 15 min washed twice and permeabilized with 0.1% (*v/v*) Triton-X100 (Sigma-Aldrich, Milan, Italy) for 10 min at room temperature. After washing with PBS, non-specific binding sites were blocked by incubation in 5 % (*w/v*) bovine serum albumin (BSA) in PBS for 30 min at room temperature. For detection of autophagy related proteins cells were incubated with LC3A/B (1:200, Cell Signaling Technology, Boston, MA, USA) overnight at 4 °C and subsequently with Goat anti-Rabbit IgG, Alexa Fluor 488 (Cat# A-11070, 1:500, Thermo Scientific, Waltham, MA, USA) for 1 h at room temperature. Cell nuclei were counterstained with Hoechst 33,342 dye (ProLong Glass Antifade Mountant with NucBlue, Thermo Scientific, Waltham, MA, USA).

### 4.8. Bioinformatics Analyses on Drugs Target Genes Prognostic Value and Differential Expression

Drugs targets gene expression and clinical data of soft tissue sarcomas (TCGA Sarcoma, SARC) were obtained from UCSC Xena Browser as TPM (Transcripts Per Million) [64]. The associations of drugs’ target-genes’ expression levels with patients’ overall and progression-free survival was estimated by log-rank test with gene-expression cut-points calculated by maximally selected rank statistics using the survminer R package [65]. Survival curves were plotted using the Kaplan–Meier method. Hazard ratios were also estimated by Cox proportional hazards regression model. By multivariate Cox proportional hazards analysis implemented in the survival package [66], we calculated the overall and the progression free survival associated to the clinical features: age, gender, histology, radiotherapy. We applied an AIC-based stepwise procedure to obtain the best candidate final Cox’s proportional hazards models.

Differential gene expression between tumor and adjacent normal tissue samples was assessed using GEPIA2 online tool, setting fold change and *p*-value cut-offs at 1.5 (Log_2_FC = 0.58) and 0.05, respectively [32]. Expression data from TCGA SARC was used to generate boxplots.

### 4.9. Statistical Analysis

The viability of cells was calculated by the normalization of absorbance values from MTS assay of treated cells against those from respective untreated controls. To calculate inhibitory concentration 50 (IC50), data were fitted with a log-logistic model using the drc R package [67]. Time effect on IC50 was also evaluated by using the compParm function.

Counts from the trypan blue assay, the flow cytometer evaluations of the apoptosis and cell cycle phases, and number of cells with abnormal nuclei under fluorescent microscopy evaluation were reported as percent of total counts in each sample. Data were reported as mean ± SE. Differences between treatments groups were estimated by paired t test, considering significant *p* values < 0.05. Analyses were performed using R software.

## 5. Conclusions

Although CHIR99021 effectiveness on ES needs to be reinforced by in-vivo models to define therapeutic doses and possible drug-combination approaches to enhance antitumoral effects [68], this work achieved two main goals. The first was to identify new drugs that could be good candidates for the treatment of epithelioid sarcoma, a rare disease in which the discovery of novel therapies is an urgent need. The second was to elucidate whether GSK-3β inhibition through CHIR99021 has antitumoral potential and to characterize its mechanism of action. In this study, we present evidence showing that CHIR99021 induces G_2_/M arrest and consequent mitotic catastrophe that ends in autophagy-mediated cell death.

## Figures and Tables

**Table 1:** Selection of the 5 drugs for which VA-ES-BJ cell line was sensitive.

Drug Name	Targets	Z Score
SB505124	TGFBR1, ACVR1B, ACVR1C	−4.42779
CHIR99021	GSK-3α, GSK-3β	−3.67765
Pevonedistat	NAE1	−2.39559
palbociclib	CDK4, CDK6	−1.36358
parthenolide	HDAC1	−0.92757

## Data Availability

Data is contained within the article or supplementary materials.

## Supplementary Materials

The following are available online at www.mdpi.com/article/10.3390/ijms222011147/s1, Figure S1: Effect of CHIR99021 on ES cells viability, Figure S2: Effects of CHIR99021 on the cell cycle distribution of dermal fibroblast cell line.
