# Supplementary material for: CHIR99021, trough GSK-3β Targeting, Reduces Epithelioid Sarcoma Cell Proliferation by Activating Mitotic Catastrophe and Autophagy
Source: Int J Mol Sci. 2021 Oct 15;22(20):11147. doi: 10.3390/ijms222011147 (PMC8538073; doi:10.3390/ijms222011147)
Supplement: Supplementary file 1 [file ijms-22-11147-s001.zip › ijms-1411268-supplementary.pdf]

International Journal of *Molecular Sciences*

MDPI

Supplementary material

CHIR99021, trough GSK-3β targeting, reduces Epithelioid Sarcoma cell proliferation by activating mitotic catastrophe and autophagy

Sabino Russi [1], Alessandro Sgambato [1], Anna Maria Bochicchio [1], Pietro Zoppoli [1], Michele Aieta [1], Alba Maria Lucia Capobianco [1], Vitalba Ruggieri [1,2], Emanuela Zifarone [1], Geppino Falco [3,4]* and Simona Laurino [1]

1 IRCCS CROB – Referral Cancer Center of Basilicata, Rionero in Vulture (PZ), Italy;
2 UOC Clinical Pathology, Altamura Hospital, Altamura (BA), Italy
3 Department of Biology, University of Naples Federico II, Naples, Italy;
4 Biogem - Istituto di Biologia e Genetica Molecolare, Ariano Irpino (AV), Italy;
* Correspondence: geppino.falco@unina.it;

Citation: Russi, S.; Sgambato, A.; Bochicchio, A.M.; Zoppoli, P.; Aieta, M.; Capobianco, A.M.L.; Ruggieri, V.; Zifarone, E.; Falco, G.; Laurino, S. CHIR99021, trough GSK-3β Targeting, Reduces Epithelioid Sarcoma Cell Proliferation by Activating Mitotic Catastrophe and Autophagy. *Int. J. Mol. Sci.* **2021**, *22*, 11147. https://doi.org/10.3390/ijms222011147

Academic Editor: Cecilia Garofalo

Received: 22 September 2021
Accepted: 12 October 2021
Published: 15 October 2021

Publisher's Note: MDPI stays neutral with regard to jurisdictional claims in published maps and institutional affiliations.

Copyright: © 2021 by the authors. Licensee MDPI, Basel, Switzerland. This article is an open access article distributed under the terms and conditions of the Creative Commons Attribution (CC BY) license (https://creativecommons.org/licenses/by/4.0/).

1.1 Supplementary Figures

A

VA-ES-BJ

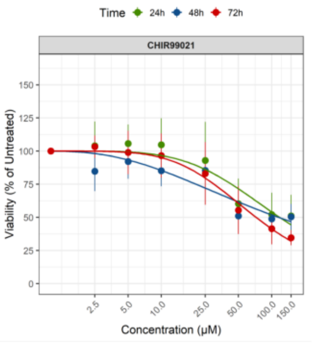

Time-effect on IC50 values

	TIME	IC50 DIFFERENCE	STANDARD ERROR	*p value*
CHIR99021	24h-48h	6.78	40.03	0.86
	24h-72h	42.02	24.82	0.09
	48h-72h	35.24	35.51	0.32

B

NEPS

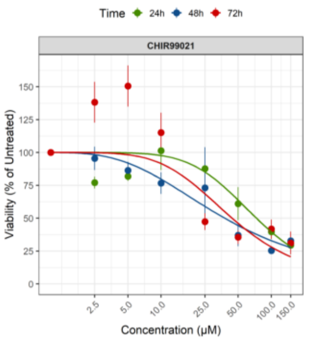

Time-effect on IC50 values

	TIME	IC50 DIFFERENCE	STANDARD ERROR	*p value*
CHIR99021	24h-48h	33.07	19.59	0.09
	24h-72h	25.67	19.77	0.19
	48h-72h	-7.40	16.11	0.64

Figure S1. Effect of CHIR99021 on ES cells viability assessed by MTS, at 24, 48 and 72h using 7 different concentrations ranging from 2.5 μM to 150 μM. (A) VA-ES-BJ and (B) NEPS cell viability was expressed as the percentage (%) of untreated cells (DMSO). The data were shown as mean and standard error of 3 independent experiments. Dose-response curves were used to estimate IC50 values of drug Tables report the time-effect on IC50 values for each cell line.

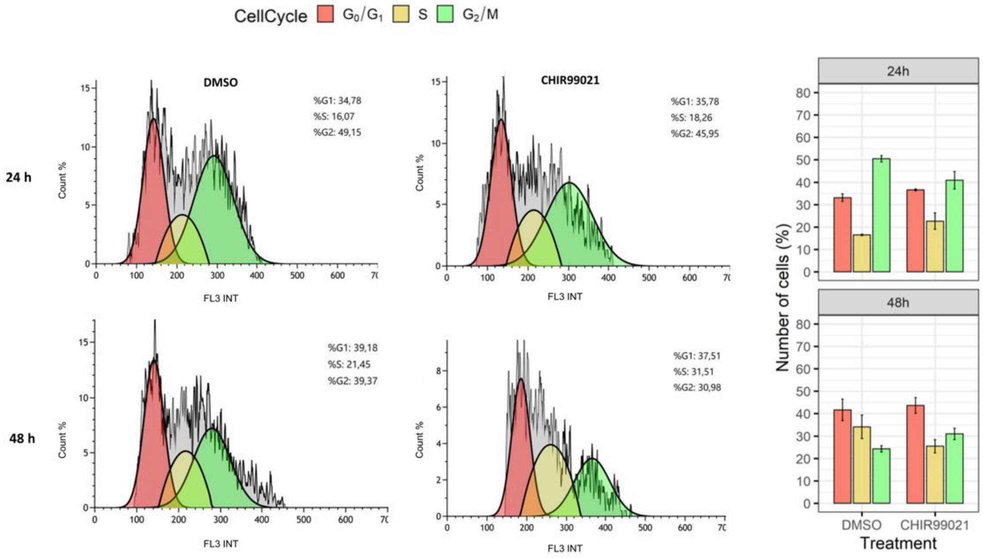

Figure S2. Effect of CHIR99021 on the cell cycle distribution of dermal fibroblast cell line. Representative plots of DNA content distribution of cell cycle phases of Primary Dermal Fibroblast; Normal, Human, Adult (HDFa) cell treated with CHIR99021 100 μM for 24 and 48h. Histogram represents the percentage of cell number in the G_0/G_1, S, and G_2/M phases after treatment, a mean of 2×10^3 events were acquired The values are representative of means ± SE of 3 separate experiments. Differences were evaluated using paired t test.
